# Non-invasive brain stimulation and pain neuroscience education in the cognitive-affective treatment of chronic low back pain: Evidence and future directions

**DOI:** 10.3389/fpain.2022.959609

**Published:** 2022-11-10

**Authors:** Cory A. Alcon, Sharon Wang-Price

**Affiliations:** ^1^ Department of Physical Therapy, High Point University, High Point, NC, United States; ^2^ School of Physical Therapy, Texas Woman’s University, Dallas, TX, United States

**Keywords:** pain catastophizing, kinesiophobia, transcranial direct current simulation, transcranial magenetic stimulation, cognition

## Abstract

Chronic low back pain (CLBP) is among the leading causes of disability worldwide. Beyond the physical and functional limitations, people's beliefs, cognitions, and perceptions of their pain can negatively influence their prognosis. Altered cognitive and affective behaviors, such as pain catastrophizing and kinesiophobia, are correlated with changes in the brain and share a dynamic and bidirectional relationship. Similarly, in the presence of persistent pain, attentional control mechanisms, which serve to organize relevant task information are impaired. These deficits demonstrate that pain may be a predominant focus of attentional resources, leaving limited reserve for other cognitively demanding tasks. Cognitive dysfunction may limit one's capacity to evaluate, interpret, and revise the maladaptive thoughts and behaviors associated with catastrophizing and fear. As such, interventions targeting the brain and resultant behaviors are compelling. Pain neuroscience education (PNE), a cognitive intervention used to reconceptualize a person's pain experiences, has been shown to reduce the effects of pain catastrophizing and kinesiophobia. However, cognitive deficits associated with chronic pain may impact the efficacy of such interventions. Non-invasive brain stimulation (NIBS), such as transcranial direct current stimulation (tDCS) or repetitive transcranial magnetic stimulation (rTMS) has been shown to be effective in the treatment of anxiety, depression, and pain. In addition, as with the treatment of most physical and psychological diagnoses, an active multimodal approach is considered to be optimal. Therefore, combining the neuromodulatory effects of NIBS with a cognitive intervention such as PNE could be promising. This review highlights the cognitive-affective deficits associated with CLBP while focusing on current evidence for cognition-based therapies and NIBS.

## Introduction

**Figure 1:**
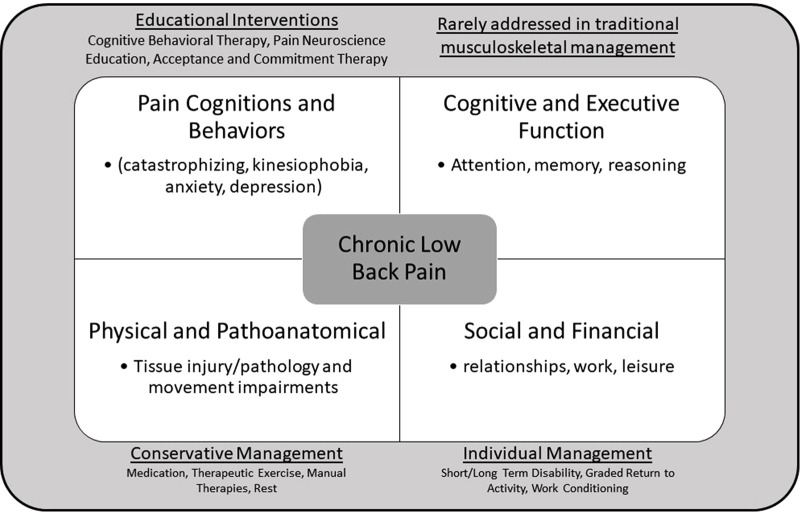
Multidimensional nature of chronic low back pain and it's traditional management approaches.

Chronic low back pain (CLBP) is among the leading causes of disability worldwide (1). Nearly one-third of the world's population lives with some form of ongoing pain, with low back and neck pain contributing the most to years lived with disability (2). This high rate of disability is associated with significant individual, social, and financial impact along with high rates of recurrence (3, 4). Despite its prevalence, CLBP often lacks a specific, identifiable cause, making it difficult to treat (5). Besides physical and functional impairments, many other factors influence the prognosis of CLBP, including a person's beliefs, cognitions, and perceptions of their pain. Those suffering from persistent pain often demonstrate altered cognitive and affective behaviors, such as pain catastrophizing, kinesiophobia, and executive control deficits (6–8). These alterations are believed to have arisen from maladaptive reorganization of brain networks, including cognitive-evaluative and affective cortical networks, and often are predictive of poorer recovery and development of chronic pain (9, 10). Elements of pain catastrophizing, fear of movement, and executive function deficits can be observed clinically through behaviors, such as magnification of pain, rumination, avoidance, withdrawal, and other adverse responses. These variables share a dynamic relationship with subjective reports of pain severity in that these behaviors can be both a consequence of pain and a predictor of chronicity. Structural and functional changes, including alterations of brain matter volume and network activation (11), also occur throughout the nervous system and correlate with the presence of pain behaviors (12). Interestingly, these cortical changes have been shown to reverse when pain is successfully treated (13). Historically, conservative management of LBP has focused on pain reduction and function improvement, with interventions targeting injured tissues taking priority (Figure 1). However, this approach could be further enhanced by also addressing coexisting psychosocial deficits (14).

As the high prevalence of CLBP continues and the evidence for the psychological contributors has grown, the effort to develop evidence-based interventions that address these behaviors has expanded. Many treatment methods are now utilized by clinicians to reduce the deleterious effects that maladaptive beliefs and behaviors can cause. Approaches such as pain neuroscience education (PNE) aim to reconceptualize a person's pain experience away from a biomedical model of pain and towards a biopsychosocial model that incorporates all facets of the pain experience. PNE consists of patient education about neurophysiology, typical pain processing, neuroplasticity, and psychosocial factors associated with acute and chronic pain. This helps patients develop more effective strategies to cope with and recover from the various dimensions of pain (15).

Considering that alterations in the cortical structure and subsequent behavioral changes are influenced by the amount of excitability within the brain regions responsible for processing the experience of pain, non-invasive brain stimulation (NIBS) techniques, such as transcranial direction current stimulation (tDCS) or repetitive transcranial magnetic stimulation (rTMS), could potentially modulate pain perception and subsequent pain behaviors (16, 17). As is the case with the treatment of most physical and psychological diagnoses, an active multimodal approach is considered optimal. Therefore, in theory, combining the neuromodulatory effects of NIBS with an active, cognitive intervention such as PNE could be promising. This review highlights cognitive-affective deficits in chronic pain, specifically CLPB, and focuses on current evidence and future directions for the therapeutic combination of cognitive therapies and NIBS for CLBP.

## Pain catastrophizing and kinesiophobia in chronic pain

Because pain is a biopsychosocial experience, the cognitive and emotional components, such as pain catastrophizing and kinesiophobia, cannot be ignored when assessing and treating patients with pain, particularly those with chronic pain. Pain catastrophizing is a maladaptive pain response characterized by rumination, helplessness and magnification regarding one's pain experience (18, 19). A person with high levels of pain catastrophizing may report feeling that their pain will continue only to worsen, progress until they are unable to function, or be caused by sinister pathology. They often have difficulty shifting their focus from painful or potentially painful stimuli and report higher threat values to non-painful stimuli (20, 21). Kinesiophobia or fear of movement is an excessive, irrational, and debilitating fear to carry out a physical movement due to a feeling of vulnerability to a painful injury or re-injury (22). Catastrophizing and kinesiophobia often coexist with an increased attentional awareness of one's pain leading to avoidance of activity based on the belief that movement will lead to further harm (23). Pain catastrophizing and kinesiophobia involve the persistence of distressing cognitive and emotional responses to pain or in anticipation of future pain, suggesting that although these behaviors can be a consequence of pain, they may also be a precursor of chronic pain (24). Evidence has shown that catastrophic thoughts and behaviors could predict the development and persistence of chronic pain (25–31). Catastrophizing also has been shown to increase attentional interference, a form of cognitive deficit, in those with chronic pain, likely a result of hypervigilance towards one's pain or in avoidance of pain that diminishes cognitive resources (32, 33).

## Cognitive deficits in chronic pain

The experience of pain is a complex cognitive process by which a person must evaluate their situation, make comparisons to previous experiences, choose a reaction, and ultimately form a mental representation of the event (34). Cognition can be defined as the procurement, processing, storage, and retrieval of information by the brain and is comprised of many factors, such as attention, perception, memory, reasoning, psychomotor skill, and executive function (35). Strong evidence has shown a close relationship between chronic pain and deficits in cognitive function, as approximately one-third of patients with chronic pain have cognitive dysfunction including difficulty with attention, learning, memory, and decision making (34, 36). Pain can be demanding of one's attention as the nervous system upregulates the amount of information needed for protection. In the presence of persistent pain, attentional control mechanisms are impaired (37). Deficits in performing tasks that require attentional shifting and the ability to selectively inhibit extraneous stimuli indicate that pain may be a predominant focus of attentional resources, leaving limited reserve for other cognitively demanding tasks (38). Similar deficits in attentional control are found in those with high pain catastrophizing (39). Impaired cognitive flexibility, attentional inhibition, attentional interference, learning, and memory are also associated with high levels of pain catastrophizing (40, 41). Cognitive dysfunction may limit one's capacity to evaluate, interpret, and revise the maladaptive thoughts and behaviors associated with catastrophizing and fear. Therefore, better understanding the cognitive profile of patients in pain can improve intervention selections and outcomes.

## Cortical changes in chronic pain

Considerable neuroanatomical and neurophysiological overlap exists between pain, emotion, and cognition. Although not clinically apparent, structural and functional changes in the brain are associated with altered cognitive and emotional processing in patients with CLBP (42). In individuals with CLBP, changes occur in areas and networks involved in the cognitive-emotional processes rather than those characteristically related to the sensory processing of pain. Specifically, the dorsolateral prefrontal cortex (DLPFC) is primarily involved in cognitive and affective processing in addition to pain processing (11). Decreased gray matter in the DLPFC has been observed in those suffering from chronic musculoskeletal pain, including LBP (43–45). Studies also have demonstrated that as levels of pain catastrophizing increase, gray matter density in the DLPFC decreases (46–48).

Moreover, the DLPFC has been shown to have a role in top-down modulation of appropriate behavioral responses (49), cognition (50), decision-making (51), and emotional control (52). Functional abnormalities involving the DLPFC are associated with the above-mentioned dysfunction in patients with depression (53), a common clinical manifestation of catastrophizing (54). Therefore, researchers proposed the abnormal functional connectivity of the DLPFC evident in patients with depression and post-traumatic disorders to be the underlying mechanism of chronic pain behaviors (i.e., catastrophizing and kinesiophobia) and cognitive deficits (42). The DLPFC is thought to serve as an interface between the three major brain networks, the resting-state default mode network (DMN), the salience network (SN) responsible for detecting behavior-relevant stimuli and allocating cognitive resources to those stimuli, and the fronto-parietal network (FPN), which coordinates behavior in a rapid, accurate and flexible goal-driven manner (11). Pain, being a relevant stimulus, typically results in the SN reducing activity of the DMN and increasing FPN activity in order to attend to the situation at hand (55, 56).

Increased DLPFC connectivity to the DMN has been observed in patients with increased levels of pain catastrophizing (45). This relationship may be partially explained by the coupling of pain and the DMN. As pain persists, it becomes part of one's identity, promotes sustained worry and fear, and progresses to functional and structural changes as the DLPFC remains activated to sustain cognitive engagement with the pain (57, 58). Further aberrant connectivity of the DLPFC to the DMN and the FPN has been shown to influence one's ability to balance cognitive demands and attention to new salient information (59, 60). For a simple task in which cognitive demand is low, patients with CLBP and high pain catastrophizing have elevated DLPFC activation, whereas healthy controls have relative deactivation (13, 47, 59). Therefore, interventions that aim at altering DLPFC activation may change pain behaviors.

## Pain neuroscience education for chronic pain

PNE is a cognitive-behavioral therapy (CBT) strategy, which aims to restructure a person's perception of pain and to promote a positive impact on the multidimensional experience of pain. When compared with other conservative strategies of pacing and self-management, participants with chronic pain who received PNE demonstrated superior knowledge of pain physiology and a significant reduction in pain catastrophizing (61). These findings have been replicated by providing participants with a booklet containing PNE metaphors and stories. Interestingly, these positive responses occurred without significant improvement in pain or self-reported disability (62, 63). PNE has also been found to reduce worry and improve physical function, mental health, and health perceptions in those diagnosed with fibromyalgia (FM) (64). When comparing PNE to biomedically focused education, small-to-moderate effect sizes were found in favor of PNE in patients with chronic spinal pain who had improved catastrophizing, kinesiophobia, and illness perceptions. However, no significance was found for perceived disability (65).

Systematic reviews support the use of PNE for musculoskeletal disorders to improve pain catastrophizing, fear-avoidance, unhealthy attitudes and behaviors, and healthcare utilization (66). While demonstrating limited short-term efficacy for measures of pain, PNE shows consistent ability to modulate the cognitive-affective domains of pain, many of which contribute to the chronicity and severity of chronic pain (67, 68). The literature on PNE contains several limitations that make results difficult to generalize, including heterogenous study designs, participant populations, outcomes measures, and PNE delivery approaches.

Neuroimaging has been used to study the underlying mechanism of CBT effects on cortical changes (71, 72). An fMRI study in FM showed that CBT normalized activation of several cortical regions related to cognitive and emotional regulations, including the DLPFC, with a concurrent reduction in depression, and anxiety symptoms (73). These fMRI results suggest that CBT could change the brain's processing of pain through increased access to executive centers for the reappraisal of pain behaviors such as catastrophizing and fear (73). The study results indicate a strong top-down control of pain, enhanced cognitive function, and altered perception of stimuli generated by CBT. To date, only two, single-subject fMRI reports have investigated the effects of PNE on brain function. Both studies showed marked differences between pre- and post-treatment fMRI scans, indicating that PNE appears to have neuromodulating effects on frontal, cingulate, and insular cortices (69, 70). Despite limited evidence, PNE, a type of CBT, could influence structural and functional connectivity changes *via* reduction of pain catastrophizing and kinesiophobia that may be occupying cognitive reserves.

## Non-invasive brain stimulation for chronic pain

tDCS and rTMS are two common NIBS techniques which have been advocated for chronic pain management although its use for chronic pain is still in the investigative phase. tDCS uses a low-intensity current that passes between two electrodes on the head, whereas rTMS uses an electromagnetic field that directs an electric current to modulate neuronal activity in targeted areas of the brain. These techniques are widely used to treat various impairments associated with depression, anxiety, stroke, spinal cord injury, Parkinson's disease, and chronic pain. Evidence also supports the use of these techniques for improving memory, attention, and learning in cognitively-impaired, and pain-suffering participants when the DLPFC was targeted (74–76). While the therapeutic mechanisms are not entirely understood, the techniques appear to be able to modulate cortical excitability and to facilitate neuroplastic changes (77–79). The effects of NIBS are shown to be related to long-term potentiation (LTP) and long-term depression (LTD)-like results depending on the direction of the tDCS current or frequency of the rTMS pulses (80, 81). Anodal tDCS leads to depolarization of the neuronal membranes that increases cortical excitability while cathodal stimulation induces hyperpolarization that decreases excitability (82). rTMS produces LTP or LTD based on pulse frequency at high (≥5 Hz) or low (≤1 Hz) frequencies respectively (78, 83–86).

Meta-analyses have demonstrated that NIBS has significant effect on pain reduction for FM, migraine, CLBP, and spinal cord injury-related pain (87–92). Studies also demonstrated that NIBS targeting the primary motor cortex (M1) had greater pain reduction than NIBS targeting the DLPFC (83–86). However, considering the cognitive-evaluative and motivational-affective domains of pain, the overlap in symptoms between those with anxiety or depression and those of chronic pain makes the DLPFC a promising therapeutic target. When targeting the left DLPFC, both tDCS and rTMS have been found to consistently and positively affect measures of depression, anxiety, and cognitive dysfunction in patients with depression (93, 94). Furthermore, NIBS targeting the DLPFC has been found to reduce depression and anxiety in patients with FM, likely as a result of targeting the two conditions that share neurological substrates. For example, a RCT showed that when tDCS targeted the DLPFC, improvements in measures of cognition and depression were superior to the intervention targeting M1 (95). Two studies investigated the influence of home-based tDCS targeting the DLPFC and showed significant improvement in pain catastrophizing, depressive symptoms, and sleep quality for FM (96, 97). Similar results have been shown following rTMS targeting the DLPFC on the affective domain of pain, including short-term improvement of depression symptoms and pain catastrophizing (98–100).

It has been speculated that the analgesic effects derived from NIBS targeting the DLPFC are the result of modulation of cognitive function (11), as DLPFC stimulation has been shown to reduce response time during working memory tasks (101) and improve sustained attention. An RCT compared effects of active vs. sham rTMS targeting the DLPFC on participants with experimentally induced elbow pain and found that participants who received active rTMS showed a trend toward improved cognitive task performance (102). In another RCT, patients with FM also demonstrated an increase of orienting and executive attentional performance following rTMS (75, 103, 104). Imaging studies suggest that tDCS to DLPFC modulates the connectivity to other areas involved in the emotional and motivational aspects of pain such as the cingulate cortex, insula, amygdala, and thalamus (105). Significant changes, including normalization of DMN and FPN connectivity have been found after anodal tDCS to the DLPFC compared to sham stimulation (106). rTMS to the DLPFC also has been shown to activate inhibitory circuits involved in pain reduction in healthy participants (107). Furthermore, higher pain thresholds and functional connectivity changes have been demonstrated with tDCS and rTMS targeting the DLPFC (108–110). These findings support that targeting the DLPFC modulates both sensory and affective networks, confirming the role of the DLPFC in pain modulation both specifically and beyond that of pain processing.

## Combined therapies: Future directions

Few studies have investigated the augmentative effect of combining NIBS with another non-pharmacological therapy. Due to its ability to alter cortical excitability, tDCS and rTMS are thought to produce a priming effect on subsequent interventions (111, 112). To date, studies have combined tDCS or rTMS to the M1 with exercise, visual illusion, and peripheral electrical stimulation (113–117). Most of these studies have shown a greater effect on pain reduction with combined interventions than isolated interventions alone (115, 116, 118, 119). Few studies (120, 121) have assessed the effects of combined NIBS with CBT for pain. However, these studies investigated either a sample of healthy participants (97) or a heterogeneous sample (98). In addition, these studies targeted the M1 for NIBS. Furthermore, these studies did not use outcome measures that can capture change of pain behaviors. A single-subject case report demonstrated that rTMS combined with CBT is a feasible intervention that significantly reduced depression (122). To date, no study has yet examined the combined effects of NIBS to the DLPFC and PNE, using outcome measures that were designed to detect changes of pain behaviors and cognition. Considering the influence of pain catastrophizing and kinesiophobia have on various domains of cognition, CBT techniques such as PNE could benefit from a precursory intervention such as tDCS or rTMS that normalize the brain function of subsequent CBT.

## Discussion

Many conservative approaches exist for the treatment of CLBP such as exercise, manual therapy, electrotherapeutic modalities, and medications. These interventions primarily focus on the injured tissues. The effects of these interventions often are small and likely due to the poorly understood mechanisms that underlie CLBP itself and typically neglect the complex cognitive and emotional factors facilitating symptom progression. However, specific assessment and determination of central nervous system mediators of pain, such as cognition/executive function, pain catastrophizing, and kinesiophobia provides insight into matching interventions with mechanisms (123, 124). Patients with CLBP who exhibit pain catastrophizing and kinesiophobia appear to be less responsive to standard, conservative interventions due to these central barriers. Therefore, approaches aimed at modulating involved brain regions, such as tDCS or rTMS, could potentially allow subsequent behavioral therapies (e.g., PNE) targeting the same regions to be more effective. Despite supporting evidence for these individual approaches, the combined effects of these two interventions have not been investigated. It remains unclear if priming the cognitive-affective circuitry that is conceptualized to support PNE with NIBS will augment the behavioral effect of PNE. However, more rigorously designed clinical trials may elucidate a novel approach to treatment of the cognitive-affective domains of pain and result in improved management of persistent pain that has grown to become one of the largest public health issues of our time.
